# Influence of protozoan grazing on magnetotactic bacteria on intracellular and extracellular iron content

**DOI:** 10.1111/1758-2229.13140

**Published:** 2023-02-13

**Authors:** Yusuke Seki, Yukako Eguchi, Azuma Taoka

**Affiliations:** ^1^ Institute of Science and Engineering Kanazawa University Kakuma‐machi, Kanazawa Ishikawa Japan; ^2^ Institute for Promotion of Diversity and Inclusion Kanazawa University Kakuma‐machi, Kanazawa Ishikawa Japan; ^3^ Nano Life Science Institute (WPI‐NanoLSI) Kanazawa University Kakuma‐machi, Kanazawa Ishikawa Japan

## Abstract

Magnetotactic bacteria (MTB) ubiquitously inhabit the oxic–anoxic interface or anaerobic areas of aquatic environments. MTB biomineralize magnetite or greigite crystals and synthesize an organelle known as magnetosome. This intrinsic ability of MTB allows them to accumulate iron to levels 100–1000 times higher than those in non‐magnetotactic bacteria (non‐MTB). Therefore, MTB considerably contributes to the global iron cycle as primary iron suppliers in the aquatic environmental food chain. However, to the best of our knowledge, there have been no reports describing the effects of trophic interactions between MTB and their protist grazers on the iron distributions in MTB grazers and the extracellular milieu. Herein, we evaluated the effects of MTB grazing using a model species of protist (*Tetrahymena pyriformis*) and a model species of MTB (*Magnetospirillum magneticum* AMB‐1). MTB‐fed *T. pyriformis* exhibited a magnetic response and contained magnetite crystals in their vacuoles. Fluorescence imaging using a ferrous ion‐specific fluorescent dye revealed that the cellular ferrous ion content was five times higher in MTB‐fed *T. pyriformis* than in non‐MTB grazers. Moreover, soluble iron concentrations in the spent media increased with time during MTB predation. This study provides experimental evidence to delineate the importance of trophic interactions of MTB on iron distributions.

## INTRODUCTION

Although iron is an essential element for virtually all organisms, it exhibits poor solubility and high toxicity. The ferric state—the predominant form of iron in aerobic environments—is extremely insoluble, and hence, biologically utilizable iron is scarce and often growth limiting in numerous ecological niches (Martin et al., 1994; Martin & Fitzwater, 1988). Bacteria have developed mechanisms of iron acquisition, storage, and detoxification and can accumulate iron from the environment. Hence, they are considered crucial for the supply of iron in food webs. In particular, magnetotactic bacteria (MTB) contain ~100–1000 times higher iron concentration per cell than that found in a non‐magnetotactic bacteria (non‐MTB) cell (Lin et al., 2017). MTB mineralize iron‐oxide or ‐sulfide magnetic crystals to form a geomagnetic sensor, which enables cells to navigate along a geomagnetic field (Bazylinski & Frankel, 2004; McCausland & Komeili, 2020; Uebe & Schüler, 2016). MTB are ubiquitous bacteria present in the sediments of freshwater, brackish, marine, and hypersaline habitats as well as in chemically stratified water columns of these environments (Lefèvre & Bazylinski, 2013; Lin et al., 2017). Studies using quantitative environmental microbiological approaches have accumulated evidence regarding MTB cell densities in environments (Bazylinski et al., 1995; Blakemore, 1982; Flies et al., 2005; Simmons et al., 2004, 2007; Spring et al., 1993). Using these insights, Amor et al. estimated that MTB can incorporate a significant fraction of the mass of dissolved iron transported to the ocean (Amor et al., 2020). The estimated mass of iron acquired by MTB corresponds to approximately 1%–500% of the mass of dissolved iron transported by the rivers to ocean annually. Therefore, MTB play a crucial role in the global hydrosphere iron cycle (Amor et al., 2020; Lin et al., 2017; Monteil & Lefèvre, 2020). However, the effects of trophic interactions of MTB on iron distributions have not been explored experimentally.

Protozoan grazing of colloidal iron has been proposed as a mechanism for generating ‘bioavailable’ iron (Barbeau et al., 1996; Sherr & Sherr, 2002). Protist predation is a major destination of bacterial cell bodies in aquatic habitats (Pernthaler, 2005) and several studies have implied that protists graze on MTB (reviewed by Monteil & Lefèvre, 2020). Bazylinski et al. found that protists accumulated magnetosome‐like iron‐containing particles within the cells (Bazylinski et al., 2000). Moreover, a moment of predation was directly observed during grazing experiments using a ciliate and the multicellular magnetotactic bacterium *Candidatus* Magnetoglobus multicellularis (Martins et al., 2007). Owing to predation, the magnetosomes of the engulfed *Ca*. M. multicellularis accumulated within the acidic vacuoles of ciliates and appeared partially digested. Monteil et al. reported an MTB‐grazing magnetic ciliate from MTB enriched environmental samples, identified as *Uronema marinum*, a known bacterivorous protist (Monteil et al., 2018). Furthermore, Chen et al. isolated the MTB‐grazing ciliate *Uronemella parafilificum* HQ from the magnetically collected sediment from the intertidal zone of Huiquan Bay (Chen et al., 2021). The ciliates contained variously shaped magnetic particles. Statistical analysis revealed that the size and shape of these engulfed magnetic crystals were similar to those in MTB present in the same environment. Their findings suggested reaffirmed the fact that protists can graze, ingest, and digest MTB. Hence, MTB predation is important for maintaining the iron cycle. However, the effects of MTB predation on iron concentrations within a protist cell and the extracellular milieu are yet to be elucidated.

In this study, we performed feeding experiments using a model species of protist (*Tetrahymena pyriformis*) and that of MTB (*Magnetospirillum magneticum* AMB‐1 [AMB‐1]). The *T. pyriformis* cells that engulfed AMB‐1 exhibited a magnetic response. The intracellular distribution of ferrous ions in *T. pyriformis* was imaged using a ferrous ion‐sensitive fluorescent dye. After feeding with AMB‐1 expressing GFP‐labelled magnetosomes, an increase in ferrous ion signals was observed in vacuoles containing the corresponding foci for GFP‐labelled magnetosomes. The cellular iron concentrations in AMB‐1‐fed *T. pyriformis* cells were five times higher than those in non‐MTB‐fed *T. pyriformis* cells. Furthermore, the dissolved iron concentration in the spent media increased significantly following the predation of AMB‐1.

## RESULTS AND DISCUSSION

### M. magneticum *AMB‐1 predation by* T. pyriformis

**FIGURE 1:**
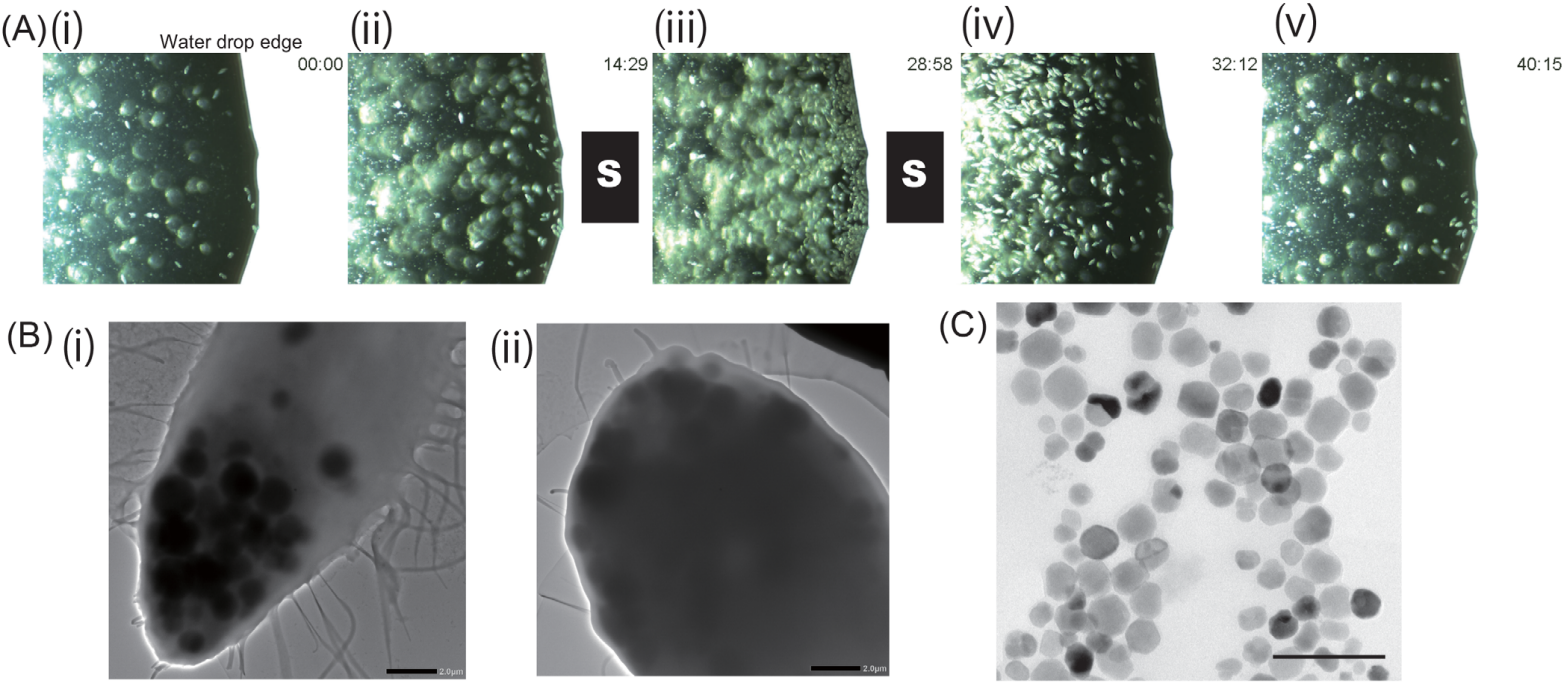
*M. magneticum* AMB‐1 engulfed by *T. pyriformis* cells. (A) Magnetic response of *T. pyriformis* cells after engulfing *M. magneticum* AMB‐1 (still images of Movie S1). The images of the edge of a water drop containing *T. pyriformis* cells 1 day post feeding with *M. magneticum* AMB‐1 cells. A bar magnet was placed beside the water drop from 10 to 30 s in Movie S1 (panels from (ii)–to (iii)). The *T. pyriformis* cells were attracted to the bar magnet. (B) Transmission electron microscopic images of *T. pyriformis* cells after engulfing *M. magneticum* AMB‐1. The cells contain electron‐dense opaque vacuoles. (C) Transmission electron microscopic images of ingested magnetosomes. Magnetosomes were collected using a bar magnet from the *T. pyriformis* cell lysate. To observe magnetosomes in *T. pyriformis* AMB‐1‐grazing cells, the cells were concentrated by centrifugation at 1,000 × *g* for 1 min and then disrupted with an ultrasonic oscillator (Branson model 450) at 20 kHz and 80 W for 30 s. The lysed cell suspension in a 15‐ml plastic tube was placed on a neodymium bar magnet for 1 h, and then the nonmagnetic fluid was removed by aspiration. The magnetically attracted magnetosomes were carefully suspended in a small amount of water and then loaded onto the surface of formvar and carbon‐coated grids, which were air‐dried. The specimens were studied under JEOL JEM 2100 Plus transmission electron microscope operating at 120 kV in the bright‐field mode. Bars: 2 μm and 200 nm in panels (B) and (C), respectively

*T. pyriformis* cells were cultured in sterilized water by feeding on non‐MTB *Pseudomonas poae* cells, used as the non‐MTB control for the feeding experiments. MTB *M. magneticum* AMB‐1 (ATCC 700264) was cultured microaerobically in *Magnetospirillum* growth media (Komeili et al., 2004) at 28°C. The bacterial cells were harvested via centrifugation at 8,000 × *g* for 10 min. The cells were then washed thrice with distilled water and suspended in sterilized water to reach a concentration of ~8 mg (wet weight)/ml both for MTB *M. magneticum* AMB‐1 (AMB‐1) and non‐MTB *P. poae*. The A_600nm_ (absorbance at 600 nm wavelength) of both suspensions was ~2.5. To feed the bacterial cells, the *T. pyriformis* culture was mixed with the same volume of bacterial cell suspension. Adding the AMB‐1 cell suspension into the *T. pyriformis* culture generated turbidity from the bacterial cells, which reduced following overnight incubation and disappeared after 3 days of incubation, indicating the grazing of AMB‐1 by *T. pyriformis* (Figure S1). We observed the co‐culture after the decreasing turbidity by a phase‐contrast microscope. Although the number of AMB‐1 cells decreased, the cells of the remaining survivors maintained cell shapes and showed motility. Therefore, the decrease in turbidity was not caused by the AMB‐1 cell lysis. *T. pyriformis* cells exhibited magnetism following overnight incubation with AMB‐1 cells. The cells in a drop of *T. pyriformis* culture were attracted to a bar magnet and accumulated at the edge of the droplet (Figure 1A, Movie S1). The *T. pyriformis* cells within the droplet moved along the magnetic fields and responded to the reversal of the magnetic field by 180° rotation, moving continuously towards the same magnetic pole (Movie S2). There was no apparent difference between the numbers of north‐ or south‐seeking cells, indicating that the cellular magnetic polarity of *T. pyriformis* was randomly obtained by the engulfed magnetosomes. This magnetic response of *T. pyriformis* cells was similar to that of a known MTB‐grazer ciliate *Uronema marinum* (Monteil et al., 2018).

Transmission electron microscopy revealed that the AMB‐1‐fed *T. pyriformis* cells contained electron‐dense opaque vacuoles (Figure 1B). Previously, in the cells of *U. marinum* that had ingested MTB, magnetosome chains containing opaque food vacuoles were observed (Monteil et al., 2018). We observed the ingested magnetosomes by magnetically collecting them from the sonicated lysate of AMB‐1‐fed *T. pyriformis* cells and found that most magnetite crystals had aggregated together; however, no chain‐like configurations were present (Figure 1C). The portion of magnetite crystals attached without spaces indicated that the magnetosome membrane and proteinaceous layer surrounding the magnetite crystals were partially digested. Martins et al. reported that magnetic particles exhibited partial digestion in the vacuoles (Martins et al., 2007). However, we did not observe obvious defects or damages in magnetite crystals in this study. Because the defected/damaged small magnetite crystals impart a weak magnetic force, they may have been removed in the magnetosome isolation step.

### Effect of AMB‐1 predation on intracellular iron concentration

**FIGURE 2:**
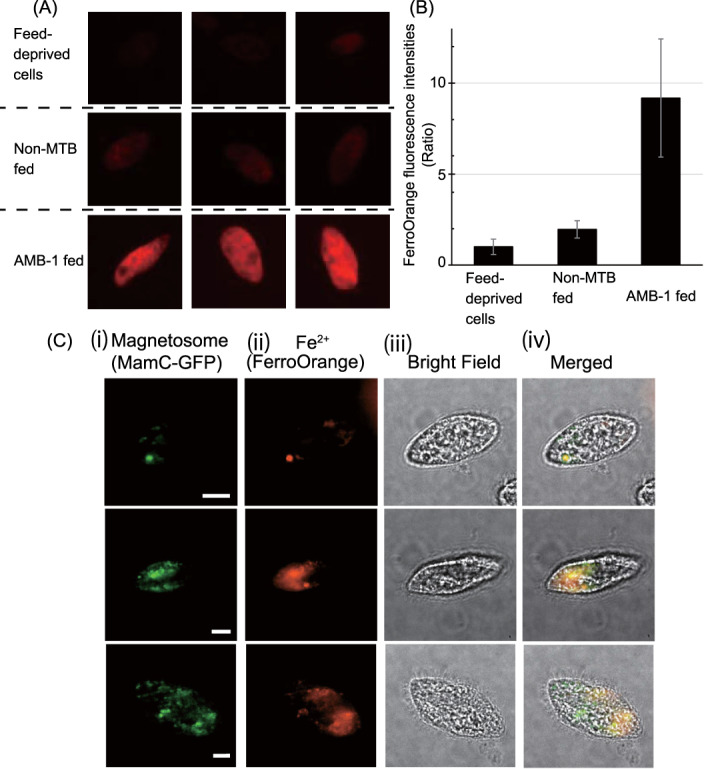
Effect of *M. magneticum* AMB‐1 predation on intracellular iron concentration. (A) Fluorescence microscopic image of FerroOrange‐treated feed‐deprived, non‐MTB‐fed, and *M. magneticum* AMB‐1‐fed *T. pyriformis* cells 27 h after the start of feeding. (B) Average fluorescence intensities of FerroOrange emitted from a single *T. pyriformis* cell of feed‐deprived (*n* = 5), non‐MTB‐fed (*n* = 18), and *M. magneticum* AMB‐1‐fed (*n* = 28) *T. pyriformis* cells 27 h from the start of feeding. Each bar represents the average fluorescence intensities, and error bars show the standard errors. (C) *T. pyriformis* cells that had engulfed MamC–GFP‐expressing *M. magneticum* AMB‐1: (i) GFP, (ii) FerroOrange, (iii) bright‐field, and (iv) merged images. Cells were chemically fixed after 5 min from the start of feeding and immediately observed under a fluorescence microscope. For fluorescence imaging, highly inclined and laminated optical sheet microscopy was used. The imaging set‐up was based on a total internal reflection fluorescence (TIRF) microscope system with an inverted microscope (Nikon, Japan), equipped with a 100× CFI Apo TIRF objective lens. The sample was illuminated with 488‐ and 562‐nm lasers (Sapphire; Coherent, USA) for GFP and FerroOrange (Dojindo, Japan; excitation/emission = 561/570–620 nm), respectively, at an inclined angle that was slightly steeper than the critical angle required for a total reflection to illuminate the entire cell. Images were acquired using a high‐sensitivity electron‐multiplying charge‐coupled device camera (iXon3; Andor, UK), with EM and preamplifier gains of 296 and 2.4×, respectively. Exposure times for the bright‐field, GFP, and FerroOrange images were 10, 300, and 900 ms, respectively. Round coverslips (Matsunami, 25‐mm diameter, 0.12–0.17 mm thick) acted as the imaging support. The coverslip was coated with poly‐l‐lysine, and 500 μl of culture was added to an Attofluor cell chamber (Thermo Fisher Scientific, USA). A 5‐mm‐thick gellan gum pad (containing 0.55% gellan gum and 0.08 mM MgCl_2_) was placed atop the coverslip to sandwich the cells against the bottom coverslip.

We examined the effect of AMB‐1 ingestion on the iron concentration of *T. pyriformis* cells using fluorescence imaging. FerroOrange is ferrous ion‐specific fluorescence reagent that enables the live‐cell imaging of ferrous ions via a specific red fluorescence. Before the feeding experiment, the *T. pyriformis* cells were treated with 1 mM 2,2'‐bipyridyl solution, a chelating agent, overnight to remove intracellular soluble ferrous ions. Before feeding bacterial cells, *T. pyriformis* cells were washed twice with water to remove 2,2'‐bipyridyl. Then, AMB‐1 and non‐MTB cells were mixed with 5 ml aliquots of *T. pyriformis* suspension to reach a final concentration of 8 mg/ml; an identical volume of water was added as a negative control (food‐deprived *T. pyriformis*). After 27 h incubation, cells were stained with 1 μM FerroOrange and observed via fluorescence microscopy. Stronger FerroOrange fluorescence signals were clearly evident in AMB‐fed cells compared with those in feed‐deprived and non‐MTB‐fed cells (Figure 2A), and the average fluorescence intensity was approximately 9‐ and 5‐fold higher than those in feed‐deprived and non‐MTB‐fed cells, respectively (Figure 2B).

Next, the intracellular localization of the ingested magnetosomes and ferrous ions were images immediately following AMB‐1 predation using 2,2'‐bipyridyl‐treated *T. pyriformis* cells fed AMB‐1 cells expressing MamC–green fluorescence protein (GFP) fusion protein, which is a magnetosome membrane protein associated with magnetite biomineralization and a maker protein for magnetite‐containing mature magnetosomes (Taoka et al., 2017). *T. pyriformis* cells were examined using GFP and FerroOrange fluorescent and bright‐field imaging 5 min after the start of feeding the AMB‐1 cells expressing MamC–GFP (Figure 2C). Spotty GFP localization suggested that AMB‐1 cells were incorporated into food vacuoles, whereas ferrous ion localization overlapped with the magnetosome GFP signal but showed a broader localization (Figure 2C). This indicates that the ferrous ions derived from AMB‐1 cells were dispersed in the *T. pyriformis* cytoplasm and that grazed MTB can be used as an iron source for phagotrophic protists. However, the number of observed GFP/FerroOrange fluorescence foci was limited to a few in the *T. pyriformis* cells, probably owing to the short feeding time (5 min), which caused the AMB‐1 cells to be engulfed by limited vacuoles in the *T. pyriformis* cells.

### Effect of AMB‐1 predation on extracellular iron concentration

**FIGURE 3:**
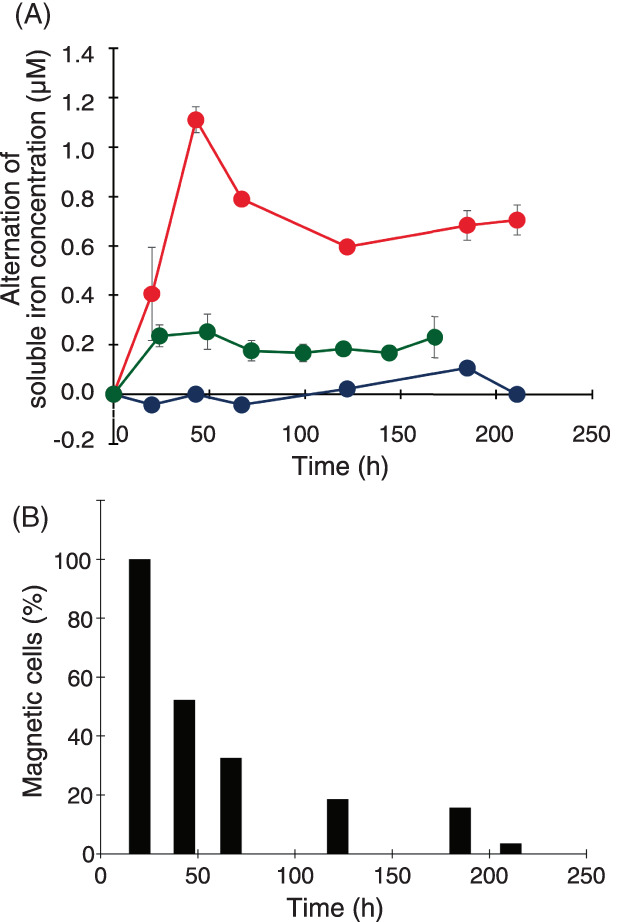
Effect of *M. magneticum* AMB‐1 predation on extracellular iron concentration. (A) Time courses of soluble iron concentration alterations after the start of cocultivations with *M. magneticum* AMB‐1 (red) and non‐MTB control cells (blue), and cultures containing only AMB‐1 without *T. pyriformis* cells (green). Values are the average of three independent cultures, and error bars show the standard errors. The iron (soluble ferric plus ferrous) concentration was measured by photometric determination using the ferrozine method (Viollier et al., 2000). Briefly, 230 μl sample or standard was added to 23 μl Reagent A (10 mM Ferrozine [Sigma‐Aldrich, USA] plus 0.1 M ammonium acetate) and the mixture was then added to 47.4 μl Reagent B (1.4 M hydroxylamine hydrochloride in a solution of 2 N HCl). The solution was incubated at room temperature for 10 min to complete the reduction of ferric ions, and then 23.7 μl Reagent C (10 M ammonium acetate buffer, pH 9.5) was added. The absorbance was measured using a plate reader (Multiskan FC, Thermo, USA) at 520 nm. A calibration curve was created using aqueous standard solutions of iron (Fujifilm‐Wako, Japan). (B) Percentages of magnetic *T. pyriformis* cells. The number of magnetic *T. pyriformis* cells was estimated by counting cells that altered their movement direction when the orientation of the bar magnet was changed by hand. Microscopic movies were obtained using an inverted phase‐contrast microscope (Eclipse Ts2, Nikon, Japan), equipped with a Moticam 3+ microscopic camera (Shimadzu Rika, Japan).

We assessed the effect of MTB predation by *T. pyriformis* on the extracellular iron concentration by comparing the alterations of soluble iron concentrations in AMB‐1‐ and non‐MTB‐fed *T. pyriformis* cultures. Figure 3A shows the time courses of soluble iron concentration alterations after the start of cocultivations with AMB‐1 and non‐MTB control cells, and cultures containing only AMB‐1 without *T. pyriformis* cells were used as a negative control. After adding AMB‐1 or non‐MTB control cell suspensions (2 mg [wet weight]/ml each) in the *T. pyriformis* cultures, a portion of the cultures (2.0 ml) was sampled at 0, 20, 43, 67, 122, 185, and 211 h following the start of bacterial feeding. The samples were centrifuged at 20,000 × *g* for 10 min to remove cells or insoluble debris. The culture supernatants obtained after centrifugation were treated with a reducing agent, hydroxylamine hydrochloride, to reduce ferric ions to ferrous ions. The soluble iron concentration (ferrous and ferric soluble ions) was measured using the ferrozine method (Viollier et al., 2000). Notably, the soluble iron concentration gradually increased to 1.1 μM during 2 days (43 h) after feeding with AMB‐1 cells and then reduced from 43 to 122 h to reach a plateau of ~0.7 μM after 122 h (Figure 3A). However, the soluble iron concentration in the culture of non‐MTB control predation remained constant, indicating that non‐MTB predation exhibited no effect on the iron concentration in the extracellular environment. We measured the alteration of soluble iron concentrations in media containing 2 mg/mL AMB‐1 cells without *T. pyriformis* cells (Figure 3A). Although the soluble iron concentration increased to 0.2 μM after 24 h incubation, the iron concentration was stably maintained at ~0.2 μM until 168 h. The soluble iron concentrations of the mixed culture containing *T. pyriformis* and AMB‐1 were two to five times higher than those containing only AMB‐1 cells for all the measuring points. This proved the contribution of AMB‐1 predation by *T. pyriformis* in elevating extracellular soluble iron concentrations. The initial iron concentration increase to 0.2 μM may be due to the lysis of unhealthy AMB‐1 cells in the cell suspension.

Recently, Amor et al. reported that a single AMB‐1 cell contains ~10^−6^ ng iron according to time‐resolved inductively coupled plasma–mass spectrometry (Amor et al., 2020). Herein, the AMB‐1 cell suspension (2 mg [wet weight] cells/ml) used as the feed for *T. pyriformis* contained 1.5 × 10^9^ AMB‐1 cells/ml as determined by direct microscopic counting in a Petroff–Hausser chamber. Based on these values, the AMB‐1 feeding culture contained 1.5 × 10^3^ ng/ml iron, representing 27 μM iron. With an approximate elevation of 1.1 μM in the soluble iron concentration during the initial 2 days of AMB‐1 predation (Figure 3A), we estimate that ~4% AMB‐1 cellular iron was released as soluble iron following AMB‐1 predation by *T. pyriformis*.

The change in proportion of magnetic *T. pyriformis* cells in the AMB‐1 feeding culture with time is shown in Figure 3B. The number of magnetic cells was estimated by counting the cells that altered their movement direction when the orientation of a bar magnet was changed. After 20 h of AMB‐1 feeding, the majority of *T. pyriformis* cells exhibited a magnetic response. However, the proportion of the magnetic cells did gradually reduce. After 43 h of feeding, the magnetic response was present in only half the cells, and after 122 h, it was retained in approximately only 20% cells. This suggests that most cells lose magnetite crystals obtained via AMB‐1 cell predation during incubation. One possibility is that the engulfed magnetite crystals are dissolved in a *T. pyriformis* vacuole, as reported for engulfed *Ca*. M. multicellularis magnetosomes that were dissolved in ciliate vacuoles (Martins et al., 2007). Another possibility is the evacuation of undigested magnetite crystals from the vacuoles. In addition, the ejection of magnetic food vacuoles from MTB‐grazing dinoflagellate protists has been previously observed (Bazylinski et al., 2000). Following the ejection, the dinoflagellate cells were no longer magnetically responsive. These suppositions require further examinations, including live‐cell imaging of the grazing process of MTB by *T. pyriformis* cells. However, according to the measurements of alterations of soluble iron concentration following MTB predation, only 4% AMB‐1 cellular iron was detected as soluble iron, indicating that a large amount of iron from AMB‐1 cells remains insoluble in the culture following MTB predation by *T. pyriformis*.

## CONCLUSION

Herein, *T. pyriformis* was shown to prey on MTB, *M. magneticum* AMB‐1. Using live‐cell imaging, ferrous ions derived from the engulfed AMB‐1 cells were detected in *T. pyriformis* cells. Furthermore, we demonstrated that the extracellular soluble iron concentration is elevated on AMB‐1 predation by the protist. We experimentally demonstrated that the iron present in MTB cells emits to the intracellular and extracellular milieu by protistan predation. This result evinces that MTB is involved as an iron supplier in global ecosystems and that the prey–predation interaction between MTB and bacterivorous protists may play a significant role on microbial ecosystems for supplying biologically utilizable soluble iron. However, the release of soluble iron to the extracellular milieu remained low at 4%, indicating that the majority of iron in AMB‐1 cells was retained in the insoluble form after *T. pyriformis* predation. It is unclear whether *T. pyriformis* is an MTB‐grazer in natural environments. Therefore, the isolation of natural MTB grazers from the environment is important to assess the significance of the iron emission found in this study. Further studies are warranted to understand to the extent of ecological impact of MTB predation on not only microbial ecology but also the wider ecosystems that encompass plants, fungi, and animals.

## AUTHOR CONTRIBUTIONS

Yusuke Seki and Yukako Eguchi performed experiments; Yukako Eguchi and Azuma Taoka analysed data; Azuma Taoka supervised the experimental design, data analysis, and data presentation; Yukako Eguchi and Azuma Taoka wrote the manuscript.

## CONFLICT OF INTEREST

The authors declare that they have no competing interests.

## Supporting information


**Movie S1:** Magnetic response of AMB‐1‐fed *T. pyriformis* cells. A bar magnet was placed on the right side of the drop of *T. pyriformis* culture at 10 s. Then, the bar magnet was removed at 30 s. Times shows s:ms.

**Movie S2:** Magnetic response of AMB‐1‐fed *T. pyriformis* cells to reversal of the magnetic fields. The bar magnet was reversed at 5, 9, 15, 18, and 23 s in this movie. The *T. pyriformis* cells responded to a reversal of the magnetic field by 180° rotation and swam continuously in the same direction towards the same magnetic pole. This movie plays at 1× speed.

**Fig. S1.** *M. magneticum* AMB‐1 predation by *T. pyriformis*. (A) Test tubes with water (blue) and *T. pyriformis* culture (pink). (B) Immediately after adding *M. magneticum* AMB‐1 cells and (C) after 3 days. The turbidity from *M. magneticum* AMB‐1 cells was reduced by *T. pyriformis* feeding.

## Data Availability

All data are available in the main text or the supplementary materials.
