# Supplementary material for: Influence of protozoan grazing on magnetotactic bacteria on intracellular and extracellular iron content
Source: Environ Microbiol Rep. 2023 Feb 13;15(3):181–7. doi: 10.1111/1758-2229.13140 (PMC10464679; doi:10.1111/1758-2229.13140)
Supplement: Supplementary file 3 — Fig. S1. M. magneticum AMB‐1 predation by T. pyriformis. (A) Test tubes with water (blue) and T. pyriformis culture (pink). (B) Immediately after adding M. magneticum AMB‐1 cells and (C) after 3 days. The turbidity from M. magneticum AMB‐1 cells was reduced by T. pyriformis feeding. [file EMI4-15-181-s001.docx]

Supporting Information for

**Influence of protozoan grazing on magnetotactic bacteria on intra- and extracellular iron content**

Yusuke Seki, Yukako Eguchi, and Azuma Taoka

**This PDF file includes:**

Figure supplements

Supplemental Figure S1

Legends for Movie S1 and S2

**Other Supplementary Materials for this manuscript include the following:**

Movie S1 and S2


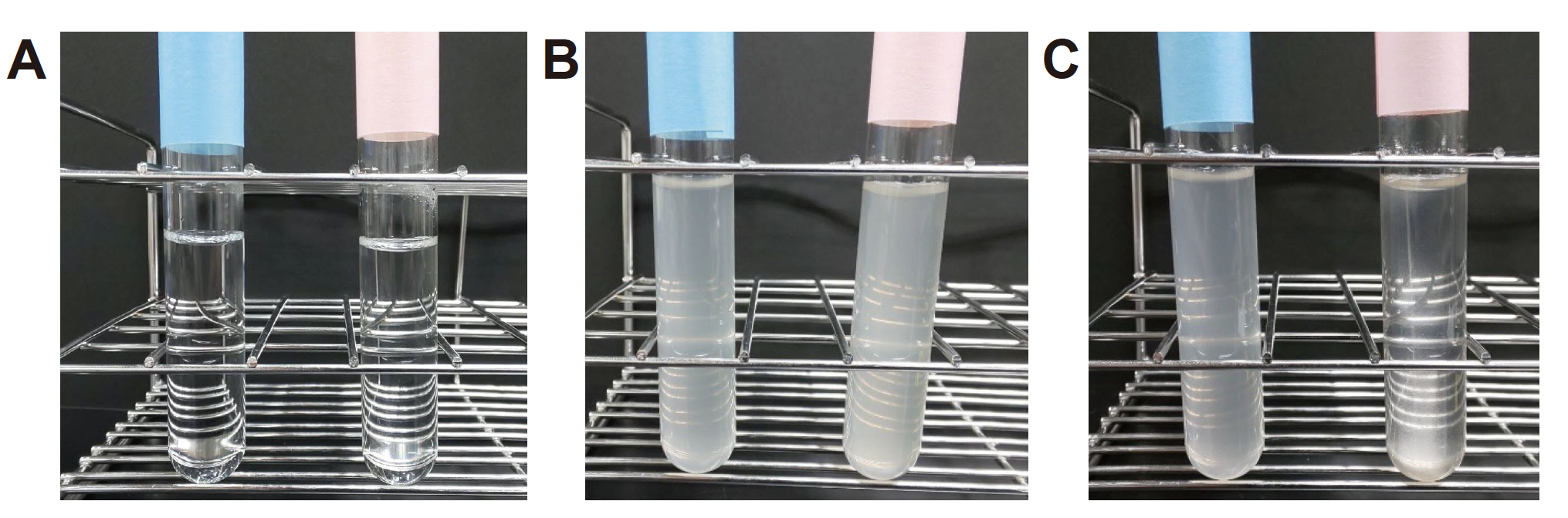


Fig. S1. *M. magneticum* AMB-1 predation by *T. pyriformis.* (A) Test tubes with water (blue) and *T. pyriformis* culture (pink). (B) Immediately after adding *M. magneticum* AMB-1 cells and (C) after 3 days. The turbidity from *M. magneticum* AMB-1 cells was reduced by *T. pyriformis* feeding.

**Legends of supplemental movies**

**Movie S1**

Magnetic response of AMB-1-fed *T. pyriformis* cells. A bar magnet was placed on the right side of the drop of *T. pyriformis* culture at 10 sec. Then, the bar magnet was removed at 30 sec. Times shows sec:msec.

**Movie S2**

Magnetic response of AMB-1-fed *T. pyriformis* cells to reversal of the magnetic fields. The bar magnet was reversed at 5, 9, 15, 18, 23 sec in this movie. The *T. pyriformis* cells responded to a reversal of the magnetic field by 180° rotation and swam continuously in the same direction toward the same magnetic pole. This movie plays at 1× speed.
